# Health-related quality of life following aortopexy for tracheomalacia: a cross-sectional study

**DOI:** 10.1093/icvts/ivae121

**Published:** 2024-07-03

**Authors:** Bethany L Brockbank, Greg S J Dewar, Richard J Hewitt, Colin R Butler, Jo Wray

**Affiliations:** UCL Great Ormond Street Institute of Child Health, London, UK; Cardiothoracic Surgery Department, Dunedin Hospital, Dunedin, New Zealand; Tracheal team, Great Ormond Street Hospital NHS Foundation Trust, London, UK; Tracheal team, Great Ormond Street Hospital NHS Foundation Trust, London, UK; Centre for Outcomes and Experience Research in Children's Health, Illness and Disability, Great Ormond Street Hospital, London, UK

**Keywords:** Aortopexy, Tracheomalacia, Paediatric, Health-related quality of life, Quality of life

## Abstract

**OBJECTIVES:**

The objective was to measure health-related quality of life (HRQoL) of children following treatment of all-cause tracheomalacia with aortopexy.

**METHODS:**

Children ≥5 years and parents of children <18 years who had undergone aortopexy completed the Paediatric Quality of Life Inventory (PedsQL4.0). Scores were compared to published norms.

**RESULTS:**

Completed questionnaires were received from 35 parents (65%) and 10 children (38%). Median age at aortopexy was 9.8 months (1 month–12.7 years), and median year of follow-up was 2.6 (4 months–6.9 years). Children who completed questionnaires had a median age of 8.4 (5.7–13.4) years. Parent- and child-reported total PedsQL scores were 69.61 (SD: 19.74) and 63.15 (SD: 20.40), respectively. Half of parents and 80% of children reported scores suggesting poor HRQoL outcomes. Parent-reported total, physical and psycho-social scores were lower than those of healthy children and those with acute illness but comparable to children with chronic health conditions and cardiovascular disease. Similarly, children themselves reported comparable total scores to children with chronic illness, but child-reported psycho-social scores were lower in the aortopexy group than any other group. There was no association between PedsQL scores and cause of malacia, age or time since aortopexy. The presence of complex congenital comorbidities had a significant (*P*<0.05) impact on HRQoL scores.

**CONCLUSIONS:**

Following aortopexy children remain at risk of poor HRQoL, especially those with complex comorbidities. HRQoL reported by both parent and child provides important insight into the lives of children following this procedure. Further longitudinal and qualitative study are required to better understand this complex group.

## Graphical Abstract

**Figure:**
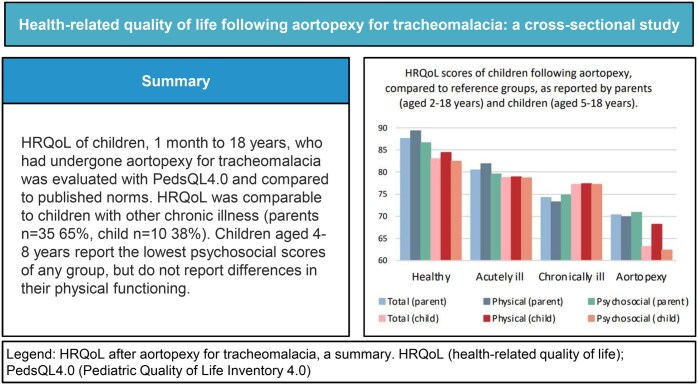

## INTRODUCTION

Tracheomalacia is the most common congenital tracheal anomaly [1]. It is characterized by increased compliance of the tracheal wall due to defects of the supportive cartilage or posterior membrane occurring in a primary form or secondary to external compression, intubation or in association with other congenital anomalies [2, 3].

The reported incidence of tracheomalacia of 1 in 1200 children [4] is likely to be an underestimation due to difficulties in diagnosis and the prevalence of mild, undetected cases. Tracheomalacia has a spectrum of presentations, from mild cough to severe, life-threatening apnoeas. It may also increase the incidence of respiratory tract infections and result in chronic lung damage [5].

Mild cases may resolve spontaneously in infancy [2] and can be managed with conservative monitoring. Medical interventions include saline nebulizers, bi-level positive airway pressure and steroids [6]. Surgical intervention with aortopexy is indicated for life-threatening events or failure to wean invasive ventilation and is generally considered the best approach if conservative measures fail [7].

Developed by Gross and Neuhauser [3], aortopexy involves lifting the aorta or other compressive vessels towards the sternum using sutures, relieving compression of the airway [8, 9]. It can be performed via sternotomy, limited sternotomy, left or right thoracotomy or thoracoscopically [9]. Satisfactory improvement in symptoms following aortopexy is reported in 80% of cases after around 3.5 years follow-up; 8% had no improvement and 4% had worsening of symptoms. There is an associated mortality (6%) and morbidity, with complications including phrenic nerve palsy and pleural effusion. Less than 1% require repeat procedures [9].

With advances in neonatal care and surgical interventions, the incidence of tracheomalacia is increasing [10], and more children are surviving following aortopexy with complex congenital anomalies and comorbidities [11]. Increasingly attention is being focused on outcomes other than those that are purely clinical and the use of patient-reported outcome measures [12] as a means of evaluating them. One patient-reported outcome is health-related quality of life (HRQoL) [13] which explores how treatments and ongoing effects of diseases and healthcare affects a child’s quality of life.

In this study, our objective was to investigate the HRQoL of children who had undergone aortopexy for the treatment of all-cause tracheomalacia at a quaternary specialist referral centre and to compare HRQoL following aortopexy with that of healthy norms and illness groups. We also aimed to evaluate the impact on HRQoL of the cause of malacia, age, time since aortopexy and the presence of comorbidities. We hypothesized that quality of life of children who had undergone aortopexy without any comorbidities would be similar to that of healthy children but that the presence of comorbidities would have a further negative impact on HRQoL.

## PATIENTS AND METHODS

### Ethical statement

This study was approved by the National Research Ethics Committee (11/LO/0133). Parents provided written consent for their participation.

### Participants

Patients with tracheomalacia treated with aortopexy at Great Ormond Street Hospital over a period of 9 years were identified via a central database. Children under 1 month and over 18 years were excluded, as were those who were deceased, living abroad or who had insufficient contact details.

### Procedure

Eligible patients were identified by a member of the clinical team and were sent written information, including age-appropriate information for children and young people aged 5 years and older, inviting them to participate in the study. They were asked to reply using a prepaid envelope if they were interested in participating. If no response was received, families were contacted by telephone to check that they had received the study information. Patient’s demographic and clinical information was extracted from the departmental database. All data were anonymized and held securely.

### Measures

HRQoL was measured using the generic Paediatric Quality of Life Inventory (PedsQL4.0) questionnaire [14, 15] for children and adolescents aged 2 to 18 years, and the PedsQL Infant Scales [16] for infants aged 1 to 23 months. This questionnaire is widely used, validated [14, 15] and quick to complete, taking approximately 5–10 min. It was completed by the parents of children aged 1 month to 18 years and by the child or young person themselves if they were 5 years or older. Different versions of the measure exist for different age groups (1–23 months, 2–4 years, 5–7 years, 8–12 years and 13–18 years). The PedsQL examines the child’s or adolescent’s quality of life in the last month. Questions cover 4 domains: physical, emotional, social and cognitive/school functioning. Scores range from 0 to 100, with higher scores indicating better HRQoL.

### Analysis

Mean subscale, summary and total scores were calculated. Means and standard deviations are reported in line with comparative published data. Statistical significance was taken as *P*-values <0.05.

Independent *t*-tests, Chi-squared or Fishers exact tests were used to compare responders with non-responders. One sample *t*-tests were used to compare total and summary (physical, psycho-social) HRQoL scores between the study cohort and published norm values for healthy, acutely ill and chronically ill children [14–16] and those with cardiovascular disease [17].

Mann–Whitney U or Kruskal–Wallis tests were used to explore the impact of the cause of malacia and presence of comorbidities on total and subgroup scores. One sample *t*-tests compared scores among different population groups. Correlations were described using Spearman Rho. Statistical analyses were undertaken using SPSS 22.0.

## RESULTS

### Participants

Eighty-two patients were identified from the database. Of those, 14 were deceased, 3 were over 18 years of age, 3 had aortopexies for tracheal stenosis, 6 lived abroad and were no longer followed at our institution and 3 had insufficient contact details. One new patient was recruited within the study period. Fifty-four patients in total were eligible and included in this study.

Of those, 28 were aged 5 and above and eligible to answer a child questionnaire, if they were able to do so. However, 2 were unable to complete the questionnaire due to having a learning disability, meaning that the final sample of children who were eligible and able to complete the questionnaire was 26. Questionnaires were received from parents of 35 children (64.8%), and from 10 children (38.4%) aged 5 and above. Demographic and clinical data are shown in Table 1.

**Table 1:** Demographic and clinical data

Variable	
Male gender, *n* (%)	21 (60)
Age of all children (years)	
Median (SD)	4.3 (3.1)
Range	0.7–12.7 years
Age of children who completed questionnaire (years)	
Median	8.4 years
Range	5.7–13.4 years
Age at aortopexy for all children (years)	
Median	9.8 months
Range	0.08–12.72 years
Re-do aortopexy procedure, *n* (%)	4 (11)
Time since aortopexy (years)	
Median	2.6 years
Range	0.3–6.9 years
Cause of tracheomalacia, *n* (%)	
Non-vascular compression	14 (40)
Vascular rings	13 (37)
Trachea-oesophageal fistula	3 (9)
Other anomalies^a^	5 (14)
Surgical approach, *n* (%)	
Limited sternotomy	22 (63)
Sternotomy	13 (37)
Comorbidities, *n* (%)^b^	
At least one	26 (74)
Cardiac^c^	21 (60)
Pulmonary^d^	16 (46)
Severe congenital^e^	18 (51)

a Including spinal deformity and mesocardia.

b Not mutually exclusive.

c Including repaired ventricular septal defects and transposition of great arteries.

d Including pulmonary atresia and pulmonary arterial hypertension.

e Excluding repaired cardiac defects.

SD: standard deviation.

Non-responders did not differ statistically from responders by gender (*P* = 0.53), cause of malacia (*P* = 0.36), presence of comorbidity (*P* = 1.00), nor specifically cardiac (*P* = 0.40), pulmonary (*P* = 0.58) or severe congenital abnormality (*P* = 0.77). There was no difference in time since last aortopexy (*P* = 0.62); however, responders were younger: 4.3 years compared to 7.3 years (*P* = 0.02) and had their last aortopexy at an earlier age: 9.8 months compared to 2.4 years (*P* = 0.04).

### Children at risk

The mean total PedsQL scores reported by parents and children were 69.61 (SD: 19.74) and 63.15 (SD: 20.40) respectively. Following aortopexy, children of all ages had lower mean HRQoL scores than healthy children, as reported by both parents and children (Table 2).

**Table 2:** Mean total scores reported by parents and children stratified by age compared to a healthy population

			Aortopexy			Healthy			% at risk	*n*
			*n*	Mean	SD	*n*	Mean	SD		
Total	Total	Parent	35	69.61	19.74					
		Child	10	63.15	20.40					
	Physical	Parent	35	69.08	26.16					
		Child	10	68.13	25.33					
	Psychosocial	Parent	35	70.34	18.26					
		Child	10	62.33	19.89					
1–12 Months	Total	Parent	3	67.82	9.11	246	82.47	9.95	100	3
	Physical	Parent	3	60.04	14.42	246	84.98	9.45	60	2
	Psychosocial	Parent	3	74.96	14.41	246	80.47	12.63	33	1
13–24 Months	Total	Parent	4	66.09	15.21	141	85.55	8.74	75	3
	Physical	Parent	4	70.12	21.58	141	88.84	7.68	75	3
	Psychosocial	Parent	4	63.22	16.61	141	83.12	11.02	75	3
2–4 Years	Total	Parent	16	77.00	18.04	2900	87.86	12.19	38	6
	Physical	Parent	16	75.59	26.20	2894	89.82	15.43	44	7
	Psychosocial	Parent	16	78.21	13.78	2898	86.56	12.31	31	5
5–7 Years	Total	Parent	6	70.91	7.86	2314	79.56	16.02	33	2
		Child	4	77.13	12.86	1915	82.22	12.55	50	2
	Physical	Parent	6	69.94	27.50	2310	80.11	20.85	33	2
		Child	4	85.94	10.67	1912	86.23	13.22	0	0
	Psychosocial	Parent	6	71.60	25.01	2314	79.25	15.44	50	3
		Child	4	72.50	15.00	1910	80.08	14.25	50	2
8–12 Years	Total	Parent	5	51.30	23.83	2935	80.48	16.28	60	3
		Child	5	50.23	19.71	2499	84.38	12.85	100	5
	Physical	Parent	5	54.38	29.11	2930	82.91	20.56	40	2
		Child	5	51.88	26.20	2494	87.98	13.77	60	3
	Psychosocial	Parent	5	49.00	22.07	2936	79.16	16.21	80	4
		Child	5	50.67	19.24	2495	82.44	14.11	80	4
13–18 Years	Total	Parent	1	54.35	^a^	1281	81.75	15.72	100	1
		Child	1	71.74	^a^	1066	85.49	12.04	100	1
	Physical	Parent	1	56.25	^a^	1279	83.87	20.13	100	1
		Child	1	78.13	^a^	1064	88.79	13.16	0	0
	Psychosocial	Parent	1	58.33	^a^	1283	80.55	15.82	80	4
		Child	1	80.00	^a^	1064	83.74	13.38	0	0

Percentage of children at risk shown.

a SD cannot be calculated as *n* = 1.

SD: standard deviation.

There were significant correlations between child- and parent-reported total (correlation coefficient 0.817, *P* = 0.04), physical (correlation coefficient 0.772, *P* = 0.009) and psycho-social scores (correlation coefficient 0.805, *P* = 0.005).

Children are considered ‘at-risk’ of poor HRQoL when their mean scores fall 1 or more SD below that of healthy norms for their age [15]. Evaluation of total scores per age group revealed that 51% of children scored below this cut-off as reported by their parent and 80% as reported by the child themselves (Table 2).

### Comparisons with other populations

**Figure 1:**
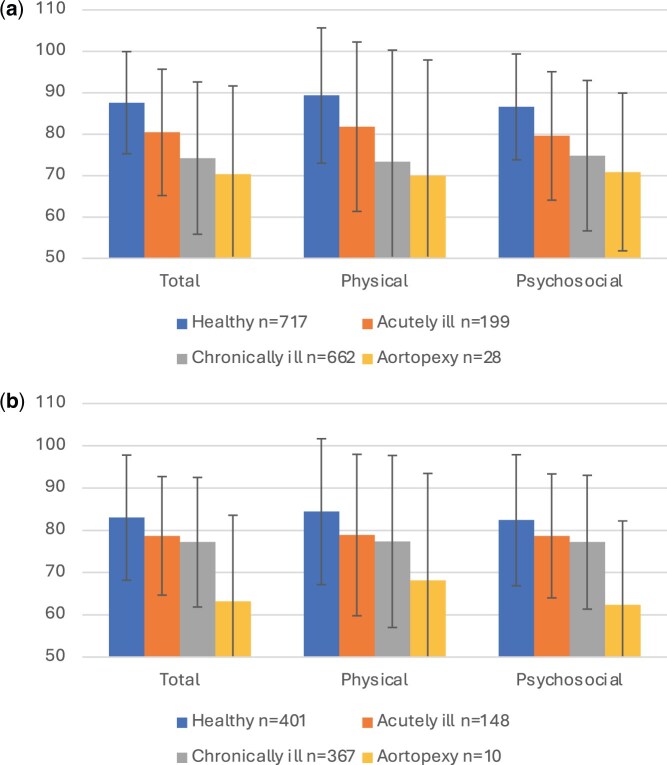
Comparison of total HRQoL scores following aortopexy with healthy, acutely and chronically ill children for (**a**) parent-reported scores, children aged 2–18 years; (**b**) child-reported scores, children aged 5–18 years. HRQoL: health-related quality of life.

Parent-proxy total, physical and psycho-social HRQoL scores for children treated with aortopexy were significantly lower than those of healthy and acutely ill populations [14–16]. There were no differences in scores compared to children with chronic illness (Table 3, Fig. 1a).

**Table 3:** Parent- and child-reported total, physical and psycho-social mean scores in aortopexy, healthy, acutely ill and chronically ill populations

	Aortopexy			Healthy			Acutely ill			Chronically ill		
	T	Ph	PS	T	Ph	PS	T	Ph	PS	T	Ph	PS
Parent score. *n *= 28												
Mean	70.30	69.90	70.86	87.61	89.32	86.58	80.42	81.81	79.56	74.22	73.28	74.80
SD	21.36	28.01	19.06	12.33	16.35	12.79	15.26	20.46	15.51	18.40	27.02	18.16
*P*-value	/	/	/	**0.000**	**0.001**	**0.000**	**0.018**	**0.033**	**0.023**	0.340	0.528	0.284
Child score. *n* = 10												
*n*	10											
Mean	63.15	68.13	62.33	83.00	84.41	82.38	78.70	78.88	78.68	77.19	77.36	77.10
SD	20.40	25.33	19.89	14.79	17.26	15.51	14.03)	19.10	14.66	15.33	20.36	15.84
*P*-value	/	/	/	**0.013**	0.073	**0.011**	**0.039**	0.212	**0.029**	0.058	0.279	**0.044**

T: total; Ph: physical; PS: psycho-social; SD: standard deviation. Bold typeface text: *p* value <0.05.

Children self-reported significantly lower total scores than their healthy and acutely ill counterparts, but there was no statistical difference in the total score compared to children with chronic illness. Physical summary scores of children with aortopexy did not differ significantly from any group, but psycho-social summary scores were significantly lower in the aortopexy group compared with all other groups (Table 3, Fig. 1b).

Parent and child scores were lower than those of age-matched children with cardiovascular disease, but these differences were only significantly different on total scores for those aged 2–4 years and self-reported total and psycho-social scores in 8–12-year-olds (Table 4).

**Table 4:** Comparison of parent- and child-reported HRQoL scores in aortopexy and cardiovascular disease populations, by age group

		Aortopexy			Cardiovascular disease			
	Age Total	*n*	Mean	SD	*n*	Mean	SD	*P*-value
Parent score	2–4 years							
	Total	16	77.00	18.04	120	86.66	12.28	0.049
	Physical	16	75.59	26.20	120	88.68	15.14	0.064
	Psychosocial	16	78.21	13.78	119	85.13	12.26	0.063
	5–7 years							
	Total	6	70.91	21.82	73	79.84	14.13	0.362
	Physical	6	69.94	33.43	73	82.44	18.25	0.402
	Psychosocial	6	71.60	17.92	73	78.45	14.75	0.649
	8–12 years							
	Total	5	51.30	23.83	142	77.98	17.27	0.067
	Physical	5	54.38	29.11	142	83.29	19.57	0.091
	Psychosocial	5	49.00	22.07	142	75.14	17.76	0.057
	13–18 years							
	Total	1	54.35	^a^	138	78.33	16.16	^b^
	Physical	1	56.25	^a^	138	81.08	17.92	^b^
	Psychosocial	1	58.33	^a^	138	76.95	17.32	^b^
Child score	5–7 years							
	Total	4	77.13	12.86	65	71.86	13.21	0.469
	Physical	4	85.94	10.67	65	80.45	16.05	0.380
	Psychosocial	4	72.50	15.00	65	67.28	14.16	0.537
	8–12 years							
	Total	5	50.23	19.71	142	78.10	15.37	0.034
	Physical	5	51.88	26.20	142	81.85	17.14	0.063
	Psychosocial	5	50.67	19.24	142	76.10	16.26	0.042
	13–18 years							
	Total	1	71.74	^a^	140	80.88	13.51	^b^
	Physical	1	78.13	^a^	140	83.17	16.57	^b^
	Psychosocial	1	80.00	^a^	140	79.63	14.44	^b^

a SD cannot be calculated as *n* = 1.

b Cannot be computed because sum of case weights equals 1.

SD: standard deviation.

### Subset analysis

**Figure 2:**
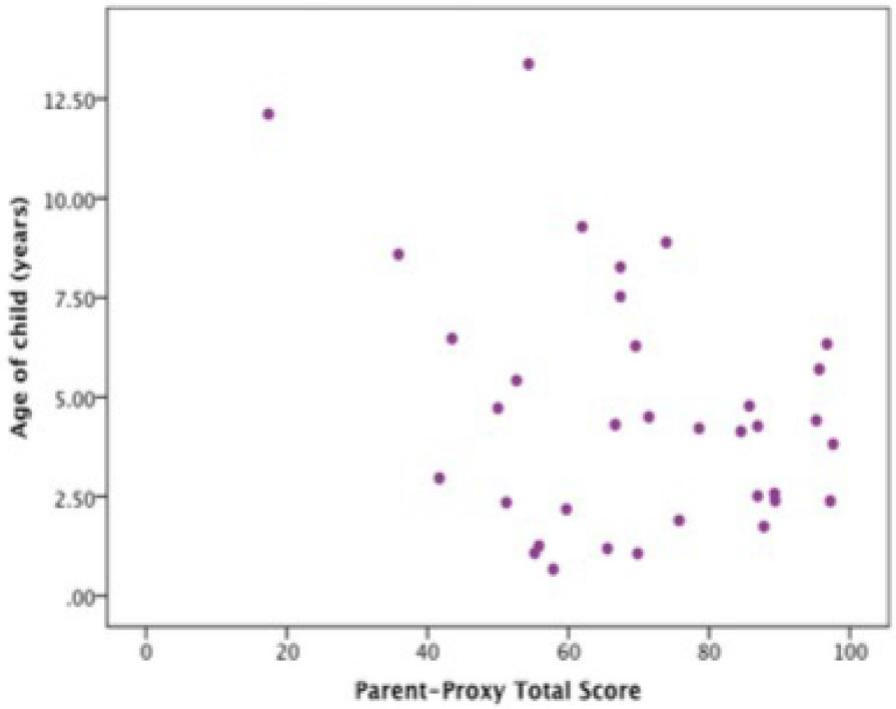
Scatter plot showing correlation between parent-reported total HRQoL score and age of child. HRQoL: health-related quality of life.

There was no significant association between any child- or parent-reported HRQoL scores and age, age at aortopexy or time since aortopexy, but there was a trend for older children to have poorer HRQoL (correlation coefficient −0.141, *P* = 0.421) (Fig. 2).

There were no differences in HRQoL scores when analysed by cause of malacia, associated pulmonary or any cardiac comorbidities for parent- or child-reported scores (*P*-values: 0.095–0.909). Children aged 2–18 years with severe congenital comorbidities had lower parent-reported total, physical and psycho-social scores than healthy norms (total score: *P* < 0.001, physical: *P* = 0.002, psycho-social: *P* < 0.001) (Table 5); however, those without significant congenital comorbidity had comparable scores to those of healthy children (total score: *P* = 0.114, physical: *P* = 0.149, psycho-social: *P* = 0.096).

**Table 5:** Parent-reported PedsQL scores for children aged 2–18 years with and without congenital comorbidities, compared with healthy norms

Scores	Healthy			Congenital abnormality							
				Yes				No			
	*n*	Mean	SD	*n*	Mean	SD	*P*-value	*n*	Mean	SD	*P*-value
Total	717	87.61	12.33	13	62.21	13.95	0.000	15	77.11	24.12	0.114
Physical	717	89.32	16.35	13	59.50	25.06	0.002	15	78.54	27.27	0.149
Psychosocial	717	86.58	12.79	13	64.62	10.88	0.000	15	76.06	22.83	0.096

SD: standard deviation.

## DISCUSSION

Aortopexy is considered the gold-standard surgical intervention for the treatment of severe tracheomalacia. Published outcomes are favourable but focus predominantly on objective surgical measures [9], and there are limited data on longer-term outcomes.

To the best of our knowledge, this is the first known exploration of the HRQoL of children following aortopexy for all-cause tracheomalacia. Results demonstrate that, despite surgical correction of their airway compromise, children still have lower HRQoL scores than a healthy reference population and those with acute illnesses. Our study found at least 50% of children are at risk of poor HRQoL outcomes, and this increases to 80% as reported by the child themselves. The effect on HRQoL seems to be ongoing, and children experience a reduction in QoL similar to those with chronic illnesses. The reduction in scores seems to be driven largely by the presence of complex congenital comorbidities.

We recognize, also, that comorbidities encompass a range of conditions, and further, multicentre study comparing the impact of various comorbidities, with and without aortopexy, could help explore these factors further.

Our data showed a trend towards older children having poorer HRQoL than younger children, as reported by their parents. A similar pattern has been shown in children following congenital cardiac surgery [17, 18]. The lower scores in older children may represent a growing awareness of a child’s limitations with age and wider disparities in health-related well-being in relation to their peers [19]. In a study of older children [20], where PedsQL was used to represent health status, this improved between the ages of 8 and 12 for both child and proxy reported scores in children following tracheo-oesophageal fistula repair, a population that will overlap with the aortopexy cohort. It is important to note that younger children made up the majority of our study population, and non-responders were statistically older. Older children are less represented in our data; therefore, further study focusing on this older child population could help us to better understand their quality of life.

While there has been shown to be good agreement between child and parent-proxy HRQoL data, both in this study and others [21], differences in the child-reported outcomes provide further interesting insights. Children, unlike their parents, report no statistical difference in physical HRQoL outcomes compared with other illness groups, and their scores are not affected by the presence of complex congenital abnormalities. Family interventions to address disparity in these perceptions can help the child’s well-being overall [22]. Children also reported worse psycho-social outcomes than all illness groups, including those with chronic illnesses. This is especially important given clinicians have been found to be poor judges of HRQoL, especially in the psycho-social domain [23], and we should therefore aim to capture this information in follow-up from the children themselves [24].

Reassuringly, HRQoL scores for children without complex congenital comorbidities were comparable to those of healthy norms. We know that following aortopexy, many children are still affected by a propensity to respiratory tract infections and, especially in those with complex comorbidities, ongoing hospitalization and medicalization may detract from both physical and emotional well-being [8, 14]. There is also wide variation in children’s and families’ responses to illness, with factors not explored in this study including anxiety being associated with poorer coping strategies and lower child HRQoL scores [25]. We recognize this is a very heterogenous group, and with relatively small numbers, subgroup analysis was limited. Further, larger-scale studies could help assess the impact, for example, of surgical outcome and redo operations.

As with many childhood diseases, there is no disease-specific HRQoL measure for this group. Identifying predictors for HRQoL outcomes is difficult [26]. The development and use of disease-specific HRQoL tools used alongside generic measures would be the gold-standard [23] approach to identifying those at risk of poor HRQoL. Using such objective patient-reported outcome measures can result in better patient-centred care [27], allow the initiation of more difficult conversations with patients and families [28], identify individualized areas for intervention and support [22] and improve HRQoL itself [29]. Given the potential for HRQoL to decline as children grow, screening for difficulties [30] may need to continue beyond the routine clinic discharge.

### Limitations

This was a single-centre study with an overall good response rate of 65% from families but a relatively low response rate from children. Its cross-sectional design captures the data at a single time-point. Absolute numbers were low, especially when groups were stratified by age or comorbidities. Due to these limitations, the calculated power of the study was low.

Questionnaires are limited by response bias; however, our analysis demonstrates the non-responders only differed statistically in age but were otherwise comparable.

The reference data set is from an American population, where toddlers under 2 years old have been shown to report lower physical and higher psycho-social HRQoL scores than their UK counterparts [31]. While this will affect the number reported at risk of poor health-related outcomes (1 SD below the norm), this affect will be minimized given that the determination of those at risk of poor health-related outcomes uses total, not subset, scores. We hope further development of UK-based reference data sets will aid further analysis in future HRQoL studies.

This is a complex cohort of children, with a spectrum of comorbidities, disease severity, symptoms and surgical interventions. Correcting for variables was not possible in this small data set. It is likely subtleties in the contributing factors to these children’s HRQoL, such as number of procedures, have not been fully appreciated.

## CONCLUSION

HRQoL is an important outcome, especially when evaluating the impact of complex procedures for complex groups of children. Our study found children with tracheomalacia following aortopexy had HRQoL outcomes not dissimilar to those with chronic illnesses, and the low HRQoL may be driven by the presence of complex congenital comorbidities. This patient group is vulnerable, with at least half of them at risk of poor HRQoL outcomes. The use of HRQoL measures may allow us to identify those at risk and offer evidence-based interventions. Disease-specific patient-reported outcome measures, along with further qualitative study, would allow us to better understand this complex group.

## Data Availability

The data underlying this article will be shared on reasonable request to the corresponding author.

## ABBREVIATIONS

HRQoL: Health-related quality of life
PedsQL4.0: Paediatric Quality of Life Inventory

## References

[1] Holinger PH , Johnston KC, Parchet VN, Zimmermann AA. Congenital malformations of the trachea, bronchi and lung. Ann Otol Rhinol Laryngol 1952;61:1159–80. 13008344 10.1177/000348945206100419

[2] Carden KA , Boiselle PM, Waltz DA, Ernst A. Tracheomalacia and tracheobronchomalacia in children and adults: an in-depth review. Chest 2005;127:984–1005. 15764786 10.1378/chest.127.3.984

[3] Gross RE , Neuhauser EBD. Compression of the trachea by an anomalous innominate artery: an operation for its relief. Am J Dis Child 1948;75:570–4. 10.1001/archpedi.1948.02030020585007 18099681

[4] Boogaard R , Huijsmans SH, Pijnenburg MWH, Tiddens HAWM, de Jongste JC, Merkus PJFM. Tracheomalacia and bronchomalacia in children: incidence and patient characteristics. Chest 2005;128:3391–7. 16304290 10.1378/chest.128.5.3391

[5] Williams SP , Losty PD, Dhannapuneni R, Lotto A, Guerrero R, Donne AJ. Aortopexy for the management of paediatric tracheomalacia—the Alder Hey experience. J Laryngol Otol 2020;134:174–7. 10.1017/S0022215120000031 31971119

[6] Fraga JC , Jennings RW, Kim PCW. Pediatric tracheomalacia. Semin Pediatr Surg 2016;25:156–64. 27301602 10.1053/j.sempedsurg.2016.02.008

[7] Gardella C , Girosi D, Rossi GA, Silvestri M, Tomà P, Bava G et al Tracheal compression by aberrant innominate artery: clinical presentations in infants and children, indications for surgical correction by aortopexy, and short- and long-term outcome. J Pediatr Surg 2010;45:564–73. 20223321 10.1016/j.jpedsurg.2009.04.028

[8] Gruszka A , Sachweh JS, Schnoering H, Tenbrock K, Muehler EG, Laschat M et al Aortopexy offers surgical options for a variety of pathological tracheal conditions in paediatric patients. Interact CardioVasc Thorac Surg 2017;25:589–94. 28605441 10.1093/icvts/ivx163

[9] Torre M , Carlucci M, Speggiorin S, Elliott MJ. Aortopexy for the treatment of tracheomalacia in children: review of the literature. Ital J Pediatr 2012;38:62. 23110796 10.1186/1824-7288-38-62 PMC3502176

[10] Hysinger EB , Panitch HB. Paediatric tracheomalacia. Paediatr Respir Rev 2016;17:9–15. 25962857 10.1016/j.prrv.2015.03.002

[11] Bové T , François K, Groote K, Suys B, de Wolf D, Verhaaren H et al Outcome analysis of major cardiac operations in low weight neonates. Ann Thorac Surg 2004;78:181–7. 15223425 10.1016/j.athoracsur.2003.12.066

[12] Black N. Patient reported outcome measures could help transform healthcare. BMJ 2013;346:f167. 23358487 10.1136/bmj.f167

[13] Drotar D. Measuring Health-Related Quality of Life in Children and Adolescents: Implications for Research and Practice. Mahwah, NJ: Lawrence Erlbaum Associates, Publishers, 1998.

[14] Varni JW , Seid M, Kurtin PS. PedsQL 4.0: reliability and validity of the Pediatric Quality of Life Inventory version 4.0 generic core scales in healthy and patient populations. Med Care 2001;39:800–12. 11468499 10.1097/00005650-200108000-00006

[15] Varni JW , Burwinkle TM, Seid M, Skarr D. The PedsQL 4.0 as a pediatric population health measure: feasibility, reliability, and validity. Ambul Pediatr 2003;3:329–41. 14616041 10.1367/1539-4409(2003)003<0329:tpaapp>2.0.co;2

[16] Varni JW , Limbers CA, Neighbors K, Schulz K, Lieu JEC, Heffer RW et al The PedsQL Infant Scales: feasibility, internal consistency reliability, and validity in healthy and ill infants. Qual Life Res 2011;20:45–55. 20730626 10.1007/s11136-010-9730-5

[17] Uzark K , Jones K, Slusher J, Limbers CA, Burwinkle TM, Varni JW. Quality of life in children with heart disease as perceived by children and parents. Pediatrics 2008;121:e1060–607. 18450848 10.1542/peds.2006-3778

[18] Tahirović E , Begić H, Tahirović H, Varni JW. Quality of life in children after cardiac surgery for congenital heart disease. Coll Antropol 2011;35:1285–90. 22397273

[19] Green A , McSweeney J, Ainley K, Bryant J. In my shoes: children’s quality of life after heart transplantation. Prog Transpl 2007;17:199–208; quiz 208. 10.1177/152692480701700307 PMC2241742 17944159

[20] ten Kate CA , Rietman AB, van de Wijngaert Y, van Gils-Frijters A, Gischler SJ, Keyzer-Dekker CMG et al Longitudinal health status and quality of life after esophageal atresia repair. J Pediatr Gastroenterol Nutr 2021;73:695–702. 34508046 10.1097/MPG.0000000000003293

[21] Varni JW , Limbers CA, Burwinkle TM. Parent proxy-report of their children’s health-related quality of life: an analysis of 13,878 parents’ reliability and validity across age subgroups using the PedsQL 4.0 Generic Core Scales. Health Qual Life Outcomes 2007;5:2. 17201923 10.1186/1477-7525-5-2 PMC1769359

[22] Mellion K , Uzark K, Cassedy A, Drotar D, Wernovsky G, Newburger JW, Pediatric Cardiac Quality of Life Inventory Testing Study Consortium et al Health-related quality of life outcomes in children and adolescents with congenital heart disease. J Pediatr 2014;164:781–8.e1. 24412135 10.1016/j.jpeds.2013.11.066

[23] Wray J , Brown K, Marino BS, Franklin R. Medical test results do not tell the whole story: health-related quality of life offers a patient perspective on outcomes. World J Pediatr Congenit Heart Surg 2011;2:566–75. 23804469 10.1177/2150135111416017

[24] Jardine J , Glinianaia SV, McConachie H, Embleton ND, Rankin J. Self-reported quality of life of young children with conditions from early infancy: a systematic review. Pediatrics 2014;134:e1129–48. https://pediatrics.aappublications.org/content/early/2014/09/17/peds.2014-0352. 25246620 10.1542/peds.2014-0352

[25] Wray J , Ryde M, Butler CR, Hewitt RJ. Quality of life can be good after slide tracheoplasty for long-segment tracheal stenosis. Interact CardioVasc Thorac Surg 2019;29:876–82. 31435669 10.1093/icvts/ivz194

[26] Montgomery J , Sau C, Clement W, Danton M, Davis C, Haddock G et al Treatment of tracheomalacia with aortopexy in children in Glasgow. Eur J Pediatr Surg 2014;24:389–93. 23918669 10.1055/s-0033-1351662

[27] Olde Rikkert MGM , van der Wees PJ, Schoon Y, Westert GP. Using patient reported outcomes measures to promote integrated care. Int J Integr Care 2018;18:8–8. 10.5334/ijic.3961 PMC6095063 30127692

[28] Varni JW , Burwinkle TM, Lane MM. Health-related quality of life measurement in pediatric clinical practice: an appraisal and precept for future research and application. Health Qual Life Outcomes 2005;3:34. 15904527 10.1186/1477-7525-3-34 PMC1156928

[29] McCusker CG , Doherty NN, Molloy B, Rooney N, Mulholland C, Sands A et al A randomized controlled trial of interventions to promote adjustment in children with congenital heart disease entering school and their families. J Pediatr Psychol 2012;37:1089–103. 22976507 10.1093/jpepsy/jss092

[30] Marino BS , Lipkin PH, Newburger JW, Peacock G, Gerdes M, Gaynor JW et al; American Heart Association Congenital Heart Defects Committee, Council on Cardiovascular Disease in the Young, Council on Cardiovascular Nursing, and Stroke Council. Neurodevelopmental outcomes in children with congenital heart disease: evaluation and management: a scientific statement from the American Heart Association. Circulation 2012;126:1143–72. 22851541 10.1161/CIR.0b013e318265ee8a

[31] Buck D. The PedsQL as a measure of parent-rated quality of life in healthy UK toddlers: psychometric properties and cross-cultural comparisons. J Child Health Care 2012;16:331–8. 23248332 10.1177/1367493512448127

